# Hypoxia Enhances Oxidative Stress in Neutrophils from ZZ Alpha-1 Antitrypsin Deficiency Patients

**DOI:** 10.3390/antiox12040872

**Published:** 2023-04-03

**Authors:** María Magallón, Silvia Castillo-Corullón, Lucía Bañuls, Daniel Pellicer, Teresa Romero, Carlos Martínez-Ferraro, María Mercedes Navarro-García, Alberto Herrejón, Cruz González, Francisco Dasí

**Affiliations:** 1 School of Medicine, Department of Physiology, University of Valencia, Avda. Blasco Ibáñez, 15, 46010 Valencia, Spain; 2 IIS INCLIVA, Rare Respiratory Diseases Group, Avda. Menéndez y Pelayo, 4, 46010 Valencia, Spain; 3 Pediatrics Unit, Hospital Clínico Universitario de Valencia, Avda. Blasco Ibáñez, 17, 46010 Valencia, Spain; 4 School of Medicine, Department of Paediatrics, University of Valencia, Avda. Blasco Ibáñez, 15, 46010 Valencia, Spain; 5 Pediatrics Unit, Hospital de Manises, Avda. Generalitat Valenciana, 50, 46940 Manises, Spain; 6 Pulmonology Unit, Hospital Doctor Peset, Avda. Gaspar Aguilar, 90, 46017 Valencia, Spain; 7 Pulmonology Unit, Hospital Clínico Universitario de Valencia, Avda. Blasco Ibáñez, 17, 46010 Valencia, Spain

**Keywords:** alpha-1 antitrypsin deficiency, chronic obstructive pulmonary disease, hypoxia, oxidative stress, cirrhosis

## Abstract

Alpha-1 antitrypsin deficiency (AATD) is a neutrophilic inflammatory disorder that may result in local hypoxia, reactive oxygen and nitrogen species (ROS/RNS) production, and increased damage in adjacent tissues. This study aims to determine the impact of hypoxia on neutrophil oxidative stress profile in AATD patients. Neutrophils were isolated from AATD patients and control volunteers and exposed to hypoxia (1% O_2_ for 4 h), ROS/RNS, mitochondrial parameters, and non-enzymatic antioxidant defenses measured by flow cytometry. The expression of enzymatic antioxidant defenses was determined by qRT-PCR. Our results indicate that ZZ-AATD neutrophils produce higher amounts of hydrogen peroxide, peroxynitrite, and nitric oxide and decreased levels of the antioxidant enzymes catalase, superoxide dismutase, and glutathione reductase. Likewise, our results show a decrease in mitochondrial membrane potential, indicating that this organelle could be involved in the production of the reactive species observed. No decrease in glutathione and thiol levels were observed. The accumulation of substances with high oxidative capacity would explain the greater oxidative damage observed in proteins and lipids. In conclusion, our results indicate that, compared to MM control individuals, ZZ-AATD neutrophils show increased ROS/RNS production under hypoxic conditions opening a new rationale for using antioxidant therapies to treat the disease.

## 1. Introduction

Alpha-1 antitrypsin deficiency (AATD) is a rare genetic condition characterized by low plasmatic levels of the protein alpha-1 antitrypsin (AAT), a protease inhibitor synthesized and secreted mainly by hepatocytes whose main function is to protect the lungs from proteolytic damage caused by proteolytic enzymes such as neutrophil elastase (NE) or proteinase-3. AAT is encoded by the *SERPINA1* gene. More than 120 mutations have been described at this locus as being the S and Z alleles (as opposed to the normal M allele), the most common deficient forms of the protein. These mutations lead to AAT polymerization, which accumulates within the hepatocytes, resulting in chronic liver inflammation and leading in some patients to the development of cirrhosis and liver cancer. Consequently, low circulating AAT levels are observed in these patients, resulting in a reduced ability to inhibit NE and leading to chronic obstructive pulmonary disease (COPD). This situation is exacerbated by smoking and repeated and prolonged exposure to dust and smoke in occupational settings and polluting environments [1,2].

However, several studies have shown that the protease–antiprotease (NE vs. AAT) imbalance is insufficient to explain the disease’s clinical variability, indicating that other modifying factors might affect the prognosis of the disease. It has been proposed that oxidative stress could be one of these factors [1]. Oxidative stress results from the imbalance between pro-oxidant and antioxidant mechanisms in the body in favor of the former. It is characterized by the accumulation of highly reactive oxygen reactive species (ROS) and reactive nitrogen species (RNS) that can react with the nucleic acids, lipids, and proteins to oxidize them, causing irreversible cell damage and leading to tissue destruction [3].

AATD is a pro-inflammatory disorder where neutrophils play a central role [4,5]. Although neutrophils participate in the organism’s defense, an excessive activation leads to an increase in the release of proteases, pro-inflammatory cytokines, and the generation of oxidative stress associated with increased damage in the adjacent tissues [6]. In this regard, it has been shown that the number of neutrophils in the lungs of patients with AATD is significantly higher than in healthy individuals, which could contribute to the increased lung damage observed in some AATD patients [5].

Under physiological conditions, circulating neutrophils are exposed to a wide range of oxygen availability, from a pO_2_ of 13 kPa in the main arteries to 3 kPa in capillaries and venules (physiological hypoxia). In addition, some tissues become profoundly hypoxic in processes such as infection or inflammation (pathological hypoxia). Therefore, neutrophils can function effectively under these conditions since tissue hypoxia is part of the normal inflammatory process [6].

Despite these observations, most of our understanding of neutrophil signaling and function comes from research done under atmospheric oxygen (21% O_2_), which does not accurately represent the physiological or pathological environment in vivo [7,8]. These observations indicate the need to study neutrophil biology under hypoxic conditions, as this is the normal environment in which they perform their functions.

Therefore, this study aimed to ascertain how hypoxia affects the neutrophil oxidative stress profile in AATD patients. We hypothesize that hypoxia will induce oxidative stress in the neutrophils of AATD patients.

## 2. Materials and Methods

### 2.1. Study Subjects

Fifty-four AATD patients and seven voluntary controls admitted from January 2018 to September 2023 to the Pediatrics Units of the Hospital Clínico Universitario Valencia (HCUV) and Hospital de Manises (Valencia, Spain) were prospectively included in the study. Patients were categorized according to their AAT phenotype. Inclusion criteria were (1) patients diagnosed with AATD according to the American Thoracic Society/European Respiratory Society recommendations [9] and (2) control individuals with MM phenotype and no history or clinical findings that suggested a pulmonary or hepatic pathology. Exclusion criteria applicable to both groups were the following: (1) cardiac dysfunction; (2) active fever or infection; (3) autoimmune diseases; (4) neurological disorders; (5) psychiatric disorders; (6) cancer; (7) treatment with antioxidants 3 months prior to sampling collection; and (8) surgery < 3 months prior to sample collection.

This study was approved by the HCUV and Hospital de Manises (Valencia, Spain) Research Ethics Committees. Procedures were explained to the children and parents, and written informed consent was obtained from parents. This study was conducted according to the Ethical Principles for Medical Research on Human Beings of the Declaration of Helsinki.

### 2.2. Anamnesis and Physical Examination

Anthropometric measurements were obtained from all the participants using standard techniques. Body mass index (BMI) was calculated as the weight (Kg)/height (m^2^) ratio. The serum concentration of AAT was measured by nephelometry. AAT phenotypes were determined by isoelectric focusing of serum samples. Pulmonary function was determined by spirometry. Liver function was assessed by measuring aspartate aminotransferase (AST), alanine aminotransferase (ALT), and γ-glutamyl transferase (GGT). Vitamin status was determined by measuring plasmatic levels of vitamins A, D, and E. Normality values are shown in the legend of Table 1.

### 2.3. Isolation and Culture of Peripheral Blood Neutrophils

Anticoagulated blood (Vacutainer tubes containing K_2_EDTA. Becton Dickinson (Franklin Lakes, NJ, USA), Cat. # 367856) was obtained from patients and healthy volunteers after 12 h of fasting. Neutrophils were isolated by negative immunomagnetic selection (EasySep™, Cat. #19666; StemCell Technologies, Vancouver, BC, USA)) according to the manufacturer’s instructions. Purified neutrophils were incubated at 9 × 10^5^/mL in RPMI (#R8758, Sigma-Aldrich, St. Louis, MO, USA) supplemented with 10% inactivated fetal bovine serum (F7524, Sigma-Aldrich), 1% sodium pyruvate (S8636, Sigma-Aldrich), and 1% non-essential amino acids (M7145, Sigma-Aldrich) for 4 h at 37 °C, as previously described [10]. To enable gas exchange, the tissue culture medium was incubated under hypoxic conditions for 3 h before being added to the tissue culture flasks.

### 2.4. Priming and Stimulation of Neutrophils

Priming of neutrophils was performed by incubation with tumor necrosis factor-alpha (TNFα) (20 ng/mL) for 30 min, followed by an activation step with the N-Formyl-L-methionyl-L-leucyl-L-phenylalanine (fMLP) (100 nM) for 10 min as previously described [10].

### 2.5. Oxidative Stress Assessment

Reactive oxygen species (ROS), reactive nitrogen species (RNS), and oxidative damage to biomolecules (lipids and proteins) were measured by flow cytometry as previously described [11,12]. Briefly, 40,000 neutrophils were incubated in 96-well plates in darkness for 20 min at 37 °C under hypoxic conditions with the appropriate probe (see Supplementary Materials, Table S1). Dead cells were excluded from the analysis by adding 4′,6-diamidino-2-phenylindole (DAPI) or propidium iodide (PI). Positive control reactions using pro-oxidant and oxidative phosphorylation uncoupling agents were performed for each marker (Supplementary Table S1).

The fluorescence generated was measured using an LSR Fortessa X-20 cytometer (Beckman-Coulter, Brea, CA, USA). The FACS Dive 4.0 software (Beckman-Coulter) was used for data acquisition, and data analysis was performed with the FLOWJO v.10.1 software (Beckman-Coulter). Results were expressed as mean fluorescence intensity in arbitrary units.

### 2.6. Expression of Antioxidant Enzymes Assessment

Gene expression analysis of the main antioxidant enzymes: Catalase, Cu/Zn superoxide dismutase (Cu/Zn SOD), Mn superoxide dismutase (Mn SOD), Glutathione peroxidase (GPx), Glutathione Reductase (GR), and the Nuclear erythroid 2-related factor (Nrf2) were performed by Real-Time Quantitative Reverse Transcription PCR (qRT-PCR). Total RNA was isolated from 7 × 10^5^ neutrophils using the NucleoSpin Triprep (#740966.50; Macherey-Nagel, Duren, Germany) following the manufacturer’s instructions. For the reverse transcription (RT), 20 ng of purified RNA was retrotranscribed with the High-capacity RNA to cDNA kit (#4387406; Thermofisher Scientific, Waltham, MA, USA) according to the manufacturer’s instructions. The cDNA was pre-amplified using gene-specific primers and probes (Thermofisher Scientific) for the main antioxidant enzymes using the pre-amplification Master Mix (#100-5744; Fluidigm, South San Francisco, CA, USA) according to the manufacturer’s instructions. Pre-amplified cDNA was amplified (PCR reaction) using the same gene-specific primers and probes used for the pre-amplification step and the TaqMan Gene Expression Master Mix (#4369016; Thermofisher Scientific) according to the manufacturer’s instructions. PCR conditions were: 2 min at 50 °C (to eliminate possible contamination from previous PCR reactions), 10 min at 95 °C for enzyme activation, followed by 2-step cycles (15 s at 95 °C; 1 min at 60 °C). The levels of B2 microglobulin were used in all samples to normalize differences in RNA input, RNA quality, and reverse transcription efficiency. Each sample was analyzed in duplicate, and the relative expression of each enzyme was calculated using the 2^−∆∆Ct^ method [13]. Therefore, final results are expressed as the fold change of each enzyme gene expression relative to the MM group (reference sample), normalized to the B2 microglobulin expression.

### 2.7. Statistical Analysis

Data are reported as mean ± standard deviation (SD). The Shapiro–Wilk normality test was used for normality assessment. Data analysis was performed using ANOVA, followed post hoc by Tukey’s multiple comparison tests in cases where the variables followed a normal distribution. The Kruskal–Wallis test was used in cases where the variables followed a different distribution from the normal one. The chi-square test was applied for proportion comparisons. The 2-tailed *p* < 0.05 was considered statistically significant. Statistical analyses were performed using GraphPad Prism 9.0 Software (GraphPad, La Jolla, CA, USA).

## 3. Results

### 3.1. Anthropometric and Clinical Data

Demographic and clinical characteristics of patients and control individuals included in this study are presented in Table 1. A total of 54 AATD patients (31 MZ, 51%; 8 SZ 13%; 15 ZZ, 25%) and 7 healthy volunteers (MM, 11%) were included in this study.

No significant differences in age, sex, BMI, and pulmonary function test or liver damage markers between the groups were observed. Similarly, no significant differences were observed in vitamin A, D, and E levels. Significant differences between groups were observed in the AAT levels (Table 1).

These results indicate that all the individuals included in this study were clinically healthy according to their physical status and the pulmonary and liver tests.

**Table 1:** Demographics and clinical characteristics of individuals included in this study.

Variable	MM (*n* = 7)	MZ (*n* = 31)	SZ (*n* = 8)	ZZ (*n* = 15)	*p*-Value
**Age (years)**	11.00 ± 5.41	7.61 ± 3.89	10.13 ± 1.25	6.69 ± 2.87	0.06
**Male/Female (%)**	43/57	52/48	75/25	54/46	0.69
**AAT (mg·dL^−1^)**	148.50 ± 18.56	85.27 ± 25.08	57.38 ± 8.89	26.22 ± 8.03	**<0.0001**
**BMI (kg·m^−2^)**	16.00 ± 2.42	17.47 ± 2.48	17.24 ± 1.41	17.50 ± 3.43	0.99
**FEV_1_ %**	-	102.50 ± 14.01	107.9 ± 5.67	102.6 ± 12.47	0.57
**FVC %**	-	98.69 ± 9.18	100.1 ± 7.60	98.44 ± 16.53	0.59
**FEV_1_/FVC %**	-	90.86 ± 6.64	94.71 ± 6.69	91.79 ± 5.22	0.22
**AST (U·L^−1^)**	20.00 ± 4.61	29.79 ± 6.57	35.00 ± 1.00	35.20 ± 6.00	0.14
**ALT (U·L^−1^)**	23.17 ± 7.65	22.76 ± 9.25	22.14 ± 4.38	32.33 ± 11.02	0.11
**GGT (U·L^−1^)**	18.46 ± 2.75	15.8 ± 2.69	17.38 ± 3.42	19.00 ± 1.00	0.14
**Vitamin A (µg·dL^−1^)**	40.10 ± 12.57	34.83 ± 11.11	32.50 ± 7.09	39.80 ± 13.94	0.69
**Vitamin D (ng·ml^−1^)**	36.50 ± 10.88	27.43 ± 9.81	35.50 ± 7.27	32.40 ± 10.46	0.18
**Vitamin E (mg·L^−1^)**	9.25 ± 3.79	10.87 ± 9.73	10.40 ± 1.14	11.72 ± 2.05	0.14

Data are presented as mean ± standard deviation. Abbreviations are as follows: **AAT**, Alpha-1 antitrypsin; **BMI**, body mass index; **FEV1**, forced expiratory volume in 1 s; **FVC**, forced vital capacity; **AST**, aspartate aminotransferase; **ALT**, alanine aminotransferase; **GGT**, gamma-glutamyl-transferase; **Normality values** are as follows: AAT: 90–100 mg/dL, FEV1: ≥ 80% predicted, FVC: ≥ 80% predicted, FEV1/FVC: ≥ 80% predicted, AST: 1–37 U/L, ALT: 1–37 U/L, GGT: 1–55 U/L, Vitamin A: 20–60 µg/dL, Vitamin D: 20–50 ng/mL, Vitamin E: 5.5–17 mg/L. *p*-values lower than 0.05 were statistically significant (labeled in bold).

### 3.2. Oxidative Stress Profile

Markers of oxidative stress, mitochondrial, intracellular calcium, and oxidative damage to biomolecule parameters are shown in Supplementary Table S2.

#### 3.2.1. Nitrogen Species Assessment

**Figure 1:**
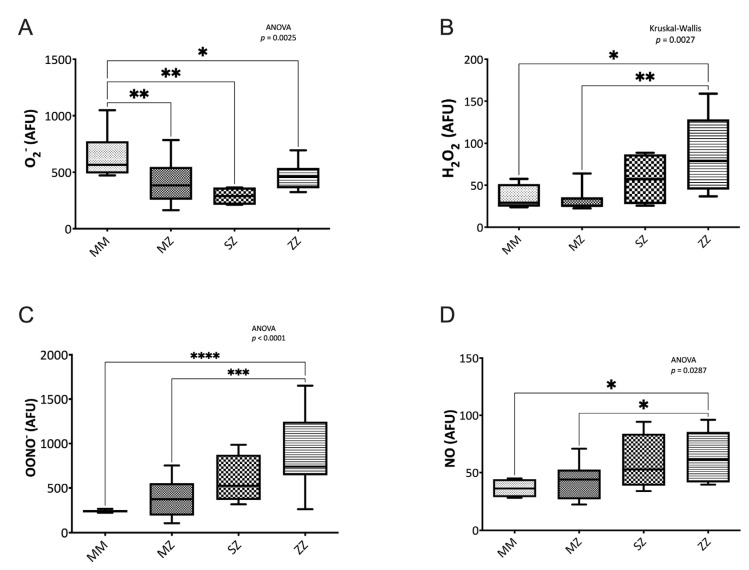
Reactive oxygen species (ROS) and reactive nitrogen species (RNS). ROS and RNS were measured by flow cytometry, as indicated in the text. Alpha-1 antitrypsin deficiency patients showed significantly decreased superoxide (O_2_^−^) levels (**A**) and increased hydrogen peroxide (H_2_O_2_) (**B**), peroxynitrite (ONOO^−^) (**C**), and nitric oxide (NO) (**D**) than control individuals. AFU: Arbitrary Fluorescence Units. Asterisks indicate the level of statistical significance: * *p* < 0.05, ** *p* < 0.01, *** *p* < 0.005, **** *p* < 0.001.

Significant differences in the oxidative stress status assessed by the O_2_^−^ (*p* = 0.002), H_2_O_2_ (*p* = 0.002), ONOO^−^ (*p* < 0.0001), and the NO (*p* = 0.028) levels were observed between groups (Figure 1). When compared to the control group, the ZZ patients showed significantly higher levels of H_2_O_2_ (*p* = 0.031), ONOO^−^ (*p* < 0–0001), and NO (*p* = 0.014) and decreased O_2_^−^ levels (*p* = 0.044). The MZ and the SZ patients also showed significantly lower levels of O_2_^−^ levels than the control group (*p* = 0.009 and *p* = 0.003, respectively). No significant differences in H_2_O_2_ (*p* > 0.999 and *p* > 0.999), ONOO^−^ (*p* = 0.623 and *p* = 0.136), and NO (*p* = 0.944 and *p* = 0.544) levels were observed between the control group and the MZ and the SZ groups. When patients’ groups were compared to each other, significant differences were observed in MZ vs. ZZ H_2_O_2_ (*p* = 0.003), ONNO^−^ (*p* = 0.0002), and NO (*p* = 0.041) levels.

#### 3.2.2. Mitochondrial Function and Intracellular Calcium Assessment

**Figure 2:**
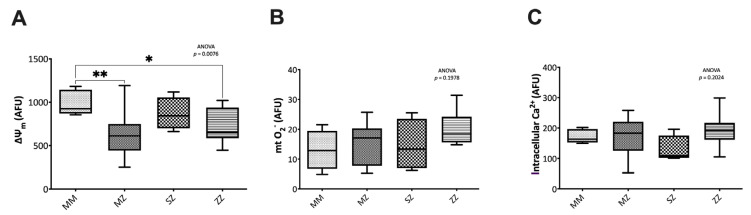
Mitochondrial function and intracellular calcium assessment. Mitochondrial membrane potential (∆ψ_m_), superoxide anion (O_2_^−^), and intracellular calcium (iCa^2+^) were measured by flow cytometry, as indicated in the text. Alpha-1 antitrypsin deficiency MZ and ZZ patients showed significantly decreased ∆ψ_m_ (**A**) than control individuals. No significant differences were observed in O_2_^−^ (**B**) and iCa^2+^ (**C**). AFU: Arbitrary Fluorescence Units. Asterisks indicate the level of statistical significance: * *p* < 0.05, ** *p* < 0.01.

Significant differences were observed in the mitochondrial membrane potential (∆ψ_m_) (*p* = 0.007). Compared to the control group, the MZ and the ZZ patients showed significantly decreased ∆ψ_m_ (*p* = 0.004 and *p* = 0.037, respectively). SZ patients also had lower ∆ψ_m_ than the control group, although the differences did not reach statistical significance (*p* = 0.9922). No significant differences in ∆ψ_m_ were observed when patient groups were compared to each other (Figure 2A). No significant differences were observed in the mitochondrial O_2_^−^ between groups (*p* = 0.1978) (Figure 2B).

Similarly, no significant differences were observed in the intracellular calcium (iCa^2+^) levels (*p* = 0.2024) (Figure 2C).

#### 3.2.3. Oxidative Damage to Biomolecules

**Figure 3:**
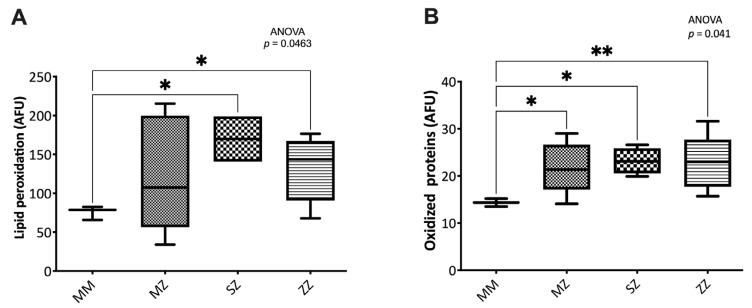
Oxidative damage to biomolecules. Protein and lipid oxidation was measured by flow cytometry, as indicated in the text. Alpha-1 antitrypsin deficiency patients showed significantly elevated oxidized proteins (**A**) and lipids (**B**) levels than control individuals. AFU: Arbitrary Fluorescence Units. Asterisks indicate the level of statistical significance: * *p* < 0.05, ** *p* < 0.01.

Significant differences were observed in oxidized proteins (*p* = 0.041) and lipid peroxidation levels (*p* = 0.046) between groups (Figure 3). The SZ and ZZ patients showed significantly increased lipid peroxidation levels (*p* = 0.0245 and *p* = 0.0283, respectively). No significant differences were observed between the MZ and the control groups (*p* = 0.3275). No significant differences were observed when patients’ groups were compared to each other (Figure 3A).

Similarly, compared to the control group, the MZ, SZ, and ZZ patients showed significantly increased levels of oxidized proteins (*p* = 0.019, *p* = 0.023, and *p* = 0.005, respectively). No significant differences were observed when patients’ groups were compared to each other (Figure 3B).

### 3.3. Antioxidant Defense Systems in AATD

The enzymatic and non-enzymatic antioxidant system results are shown in Supplementary Table S3.

#### 3.3.1. Expression of Antioxidant Enzymes

**Figure 4:**
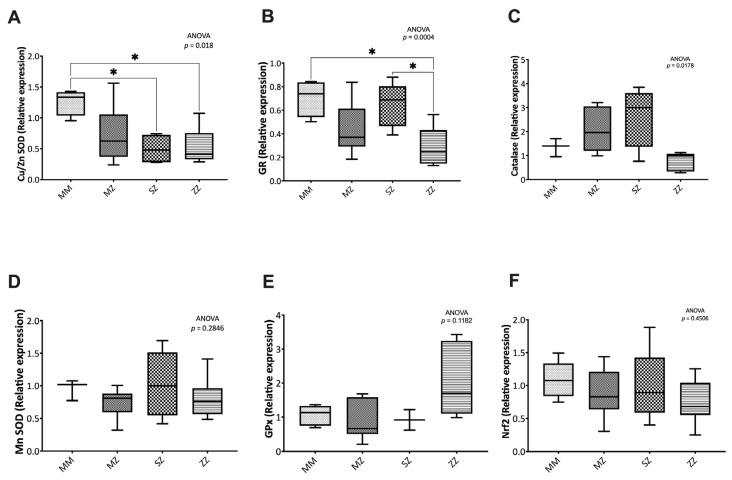
Relative expression of the main antioxidant enzymes and the Nrf2 factor. Relative expression of Cu/Zn Superoxide Dismutase (Cu/Zn SOD) (**A**), Glutathione Reductase (GR) (**B**), Catalase (**C**), MnSOD (**D**), Glutathione Peroxidase (GPx), (**E**) and Nuclear erythroid 2-related factor (Nrf2) (**F**) were determined by qRT-PCR, as indicated in the text. Asterisks indicate the level of statistical significance: * *p* < 0.05.

Significantly decreased Cu/Zn SOD and GR levels were observed between groups (*p* = 0.018 and *p* = 0.004, respectively) (Figure 4). Compared to the control group, the MZ, SZ, and ZZ patients showed significantly decreased levels of Cu/Zn (*p* = 0.1211, *p* = 0.013, and *p* = 0.014, respectively). When patients’ groups were compared to each other, no significant differences were observed (Figure 4A).

Significantly decreased levels of GR were observed between the ZZ and the control group (*p* = 0.014). The SZ and the MZ groups also showed lower levels of GR expression, although the results did not reach statistical significance (*p* = 0.103 and *p* = 0.633, respectively) (Figure 4B).

No significant differences were observed in catalase (*p* = 0.1782, Figure 4C), MnSOD (*p* = 0.2846, Figure 4D), GPx (*p* = 0.1182, Figure 4E), and Nrf2 (*p* = 0.4506, Figure 4F).

#### 3.3.2. Non-Enzymatic Antioxidant Systems

**Figure 5:**
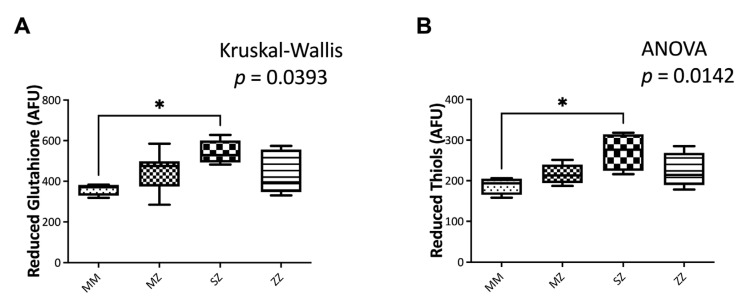
Non-enzymatic antioxidants in AATD patients. Reduced glutathione (**A**) and reduced thiols (**B**) were measured by flow cytometry, as indicated in the text. Asterisks indicate the level of statistical significance: * *p* < 0.05.

Significant differences in GSH (*p* = 0.039) and reduced thiols (*p* = 0.014) between groups (Figure 5) were observed. Compared to the control group, SZ patients showed significantly elevated GSH levels (*p* = 0.034). No significant differences were observed between the MZ (*p* = 0.758), the ZZ (*p* > 0.999), and the control group. No significant differences were observed when patients were compared to each other (Figure 5A).

Similarly, SZ patients showed significantly elevated GSH levels (*p* = 0.011) (Figure 5B) compared to the control group. No significant differences were observed between the MZ (*p* = 0.294), the ZZ (*p* = 0.189), and the control group. No significant differences were observed when patients were compared to each other (Figure 5B).

## 4. Discussion

The ability of neutrophils to damage adjacent tissues is related to their activation state. Neutrophils are cells involved in the innate or nonspecific immune response and are the first cell type to reach sites of infection and inflammation, where they play a crucial role in host defense against microbial pathogens [6]. However, excessive or uncontrolled activation of neutrophils leads, on the one hand, to their degranulation and the release of proteolytic enzymes, whose accumulation in the extracellular space results in increased damage to adjacent tissues [6]. On the other hand, activated neutrophils increase their oxygen consumption which leads (through NADPH oxidase) to the generation of ROS and RNS, which, together with SOD, myeloperoxidase, H_2_O_2,_ and hypochlorous acid, contributes to decreasing local O_2_ levels, creating a situation of local hypoxia and oxidative damage in the affected tissues [6]. Reactive oxygen and reactive nitrogen species are involved in various neutrophil processes beyond the oxidative burst, including modulation of the immune response, chemotaxis, adhesion, rolling, phagocytosis, and neutrophils extracellular traps (NETs) formation [14]. However, reactive species must be in balance with the antioxidant system since an excessive or insufficient presence of these species can lead to oxidative or reductive stress, which may affect cell physiology.

Beyond its antiprotease activity, AAT is an anti-inflammatory protein. Inflammation is accompanied by severe hypoxia within the affected tissue and is characteristic of various respiratory processes, such as COPD, cystic fibrosis, asthma, and AATD. Therefore, AATD is considered an inflammatory disease nowadays [4,5,15]. Several studies have demonstrated that the molecular mechanisms controlling inflammation and hypoxia interact significantly. A rise in plasma levels of pro-inflammatory cytokines (IL-6, TNF-alpha, and IL-1) was observed in mice subjected to hypoxic conditions [16]. Similarly, after a 3-day stay at a high altitude, healthy participants showed increased IL-6, IL-1RA, and C-reactive protein plasma levels [17]. Acute respiratory distress syndrome [18], COPD [19], and cystic fibrosis [20] are only a few of the acute and chronic respiratory disorders where this inflammation–hypoxia relationship has been reported so that airway-infiltrating neutrophils in these individuals may directly contribute to the hypoxia of affected tissues. Neutrophilic airway inflammation has been linked to both cystic fibrosis disease development and a decline in lung function in COPD patients [19,20].

Since tissue hypoxia is a natural component of inflammation, neutrophils, and macrophages, which are the cells that initiate the inflammatory response, have developed cellular and molecular mechanisms that enable them to operate properly in low-oxygen environments [6,21]. In addition, neutrophils are short-lived cells that easily undergo apoptosis in homeostasis to prevent degranulation, the release of proteinases, and subsequent cell damage. Nevertheless, neutrophil survival is greatly increased under hypoxic settings, which is likely to delay the healing of inflammation and accelerate tissue damage, possibly by causing the production of proteases and ROS [6,22]. In fact, it has been shown that the number of neutrophils found in the lungs of AATD patients is significantly increased, which will further contribute to a decrease in local O_2_ levels, increasing ROS/RNS production and leading to oxidative damage to biomolecules (DNA, lipids, and proteins) that will contribute to the development of lung tissue damage.

Overall, these observations indicate that oxidative stress may play an important role in neutrophil physiology in AATD patients. However, most studies have been performed under normoxic conditions (21% O_2_), which may not adequately reflect neutrophils’ physiological and pathophysiological conditions. Therefore, in this study, we set out to study the role of hypoxia in producing ROS/RNS by neutrophils from patients with AATD. Since, as previously mentioned, AATD is an inflammatory disease in which neutrophils play an important role, we hypothesized that local hypoxia associated with the inflammatory process would produce an increase in the generation of ROS/RNS by neutrophils [5].

Our results support that hypothesis. Increased oxidative stress in neutrophils from AATD patients, particularly those with the ZZ phenotype, is observed. Increased H_2_O_2_ levels in neutrophils isolated from asymptomatic ZZ children are observed. Accordingly, our results show a decreased expression of the antioxidant enzyme catalase in ZZ neutrophils. Catalase’s main function is the elimination of H_2_O_2_ in water and oxygen, thereby keeping the cell’s concentration of the molecule at its optimal level, which is necessary for cellular signaling activities. Low catalase levels would explain the H_2_O_2_ accumulation, which will induce the expression of glutathione peroxidase (GPx), a selenium-containing antioxidant enzyme that effectively reduces H_2_O_2_ and lipid peroxides to water and lipid alcohols.

Similarly, increased ONOO^−^ and NO levels are observed in ZZ-AATD patients. High NO levels will outcompete endogenous levels of superoxide dismutase. NO will react quickly with O_2_^−^ to form ONOO^−^ an oxidant molecule that interacts with nucleic acids, lipids, and proteins via direct oxidative reactions or by radical-mediated mechanism [23,24].

A reduction in glutathione reductase (GR) expression was observed in neutrophils from ZZ children compared with MM controls. GR catalyzes the reduction of glutathione disulfide (GSSG) to reduced glutathione (GSH), one of the most abundant reducing thiols in most cells. Surprisingly, our results do not show a decrease in GSH levels, which could be explained as a GSH de novo synthesis. The maintenance of physiological GSH and reduced thiol levels could be explained as an adaptive mechanism to the increased oxidative stress observed.

Since mitochondria are one of the main ROS sources, we decided to investigate their role in producing reactive species in our study. Our study shows a decrease in mitochondrial membrane potential in ZZ patients, suggesting that this organelle could be involved in the increased production of reactive species observed.

In our study, we analyzed the levels of vitamins A, D, and E since they have an antioxidant capacity, and their deficiency could modify the oxidative profile of neutrophils. No significant differences were observed in the levels of these vitamins, indicating that a vitamin deficit is not the cause of increased oxidative stress.

The accumulation of substances with high oxidative capacity, such as hydrogen peroxide and peroxynitrite, would explain the increased oxidative damage to the biomolecules, as confirmed by increased carbonylated proteins (MZ, SZ, and ZZ) and lipid peroxidation levels (ZZ) in neutrophils from AATD children.

Our results are in agreement with previous studies. Several groups have shown that oxidative stress contributes to liver and lung damage development in animal models of AATD [25,26,27]. Our research group has previously shown that oxidative stress produced by reduced antioxidant defenses is involved in the AATD pathophysiology and is associated with an increased risk of developing lung and/or liver disease. Our studies have shown that patients with intermediate (SZ phenotype) and high (ZZ phenotype) risk of developing emphysema and/or liver disease related to AATD showed significantly higher levels of oxidative damage markers in DNA (8-OHdG), lipids (MDA), and oxidized proteins (carbonylation). Compared to the control group, the intermediate- and high-risk groups showed significantly lower levels of total glutathione and reduced glutathione, a decrease in catalase activity, leading to an accumulation of H_2_O_2_, which would explain the significantly increased levels of oxidative stress markers observed in these patients. Additionally, a gradation in oxidative stress parameters was observed when patients were compared with each other, as the expression of the Z allele produces a higher oxidative stress state in homozygous (ZZ) than heterozygous (MZ; SZ) patients [28]. Subsequent studies by our research group demonstrated that AATD patients have significantly shorter telomeres than control individuals [29]. Recently, our group has shown that replacement therapy initially (day 2 after infusion) decreases H_2_O_2_ levels in ZZ patients. This situation is reversed 7 days after the initial AAT infusion, where H_2_O_2_ levels increase again, suggesting a possible role in oxidative stress regulation of replacement therapy [30]. These data suggest that oxidative stress could play an important role in the decline of lung and/or liver function in AATD, a situation that could be reversed by replacement therapy.

Our study has several limitations that we would like to point out. The greatest limitation is the low number of patients with the SZ and ZZ phenotypes, which is not surprising given the low prevalence of these phenotypes diagnosed. The small sample size of each group could pose a problem in reaching definitive conclusions. However, the significant differences observed in some of the analyzed parameters indicate that the results should not vary significantly after increasing the sample size. Possible sex/gender differences were not examined for the same reason. One of the main advantages of this study is that neutrophils were obtained from clinically healthy AATD children. All patients included in this study had normal liver enzyme makers, normal respiratory function (assessed by spirometry performed in this study and previous X-ray and CT scans that revealed no signs of lung damage), and normal liver function tests. Since children do not use tobacco or alcohol, the bias caused by these factors can be avoided, allowing the findings to be attributed to AATD and not to the impact of confounding factors.

As far as we know, this is the first time that the role of hypoxia in neutrophil oxidative stress in AATD patients has been investigated. Since hypoxia is involved in the pathophysiology of many respiratory diseases, including COPD, cystic fibrosis, and asthma, basic research into hypoxia can reveal new cellular processes that could be used to develop new therapeutic strategies.

The main contribution of this study to current knowledge on AATD neutrophil oxidative stress production relies upon the effects of hypoxia, which induces an imbalance in the oxidative stress equilibrium in ZZ-AATD patients, suggesting that neutrophils of these patients have a greater capacity to damage adjacent tissues.

## 5. Conclusions

At this point, we would like to emphasize that this study was not designed to evaluate the differences between hypoxia and “normoxia” but to assess the effect of hypoxia on the AATD neutrophils, as this is a neutrophils’ physiological situation and is also the situation in which some AATD patients find themselves in more advanced stages of the disease. Our study is important since it has been shown that cell cultures in hyperoxia fail to reproduce physiological and pathological conditions in vivo, even altering cellular response to drugs and hormones. In conclusion, our results indicate that, compared to MM control individuals, ZZ-AATD neutrophils show increased ROS/RNS production under hypoxic conditions opening up a new rationale for using antioxidant therapies to treat the disease.

## Data Availability

The data may be provided upon request to the principal investigator as long as the motivation is duly justified.

## Supplementary Materials

The following supporting information can be downloaded at: https://www.mdpi.com/article/10.3390/antiox12040872/s1, Table S1: Summary of flow cytometry methods; Table S2: Oxidative stress, mitochondrial, intracellular calcium, and oxidative damage parameters of neutrophils isolated from individuals included in the study; Table S3: Enzymatic and non-enzymatic defense mechanisms of neutrophils isolated from individuals included in the study.

## References

[1] Torres-Durán M., Lopez-Campos J.L., Barrecheguren M., Miravitlles M., Martinez-Delgado B., Castillo S., Escribano A., Baloira A., Navarro-Garcia M.M., Pellicer D. (2018). Alpha-1 Antitrypsin Deficiency: Outstanding Questions and Future Directions. Orphanet. J. Rare. Dis..

[2] Strnad P., McElvaney N.G., Lomas D.A. (2020). Alpha1-Antitrypsin Deficiency. N. Engl. J. Med..

[3] Palacio P.L., Godoy J.R., Aktas O., Hanschmann E.-M. (2022). Changing Perspectives from Oxidative Stress to Redox Signaling—Extracellular Redox Control in Translational Medicine. Antioxidants.

[4] Belchamber K.B.R., Walker E.M., Stockley R.A., Sapey E. (2020). Monocytes and Macrophages in Alpha-1 Antitrypsin Deficiency. Int. J. Chronic. Obstr..

[5] McCarthy C., Reeves E.P., McElvaney N.G. (2016). The Role of Neutrophils in Alpha-1 Antitrypsin Deficiency. Ann. Am. Thorac. Soc..

[6] Williams A.E., Chambers R.C. (2016). Neutrophils and Tissue Damage: Is Hypoxia the Key to Excessive Degranulation?. Thorax.

[7] Stuart J.A., Fonseca J., Moradi F., Cunningham C., Seliman B., Worsfold C.R., Dolan S., Abando J., Maddalena L.A. (2018). How Supraphysiological Oxygen Levels in Standard Cell Culture Affect Oxygen-Consuming Reactions. Oxid. Med. Cell. Longev..

[8] Alva R., Gardner G.L., Liang P., Stuart J.A. (2022). Supraphysiological Oxygen Levels in Mammalian Cell Culture: Current State and Future Perspectives. Cells.

[9] Miravitlles M., Dirksen A., Ferrarotti I., Koblizek V., Lange P., Mahadeva R., McElvaney N.G., Parr D., Piitulainen E., Roche N. (2017). European Respiratory Society Statement: Diagnosis and Treatment of Pulmonary Disease in A1-Antitrypsin Deficiency. Eur. Respir. J..

[10] Hoenderdos K., Lodge K.M., Hirst R.A., Chen C., Palazzo S.G.C., Emerenciana A., Summers C., Angyal A., Porter L., Juss J.K. (2016). Hypoxia Upregulates Neutrophil Degranulation and Potential for Tissue Injury. Thorax.

[11] Beltrán B., Nos P., Dasí F., Iborra M., Bastida G., Martínez M., O’Connor J., Sáez G., Moret I., Ponce J. (2010). Mitochondrial Dysfunction, Persistent Oxidative Damage, and Catalase Inhibition in Immune Cells of Naïve and Treated Crohn’s Disease. Inflamm. Bowel. Dis..

[12] Reula A., Pellicer D., Castillo S., Magallón M., Armengot M., Herrera G., O’Connor J.-E., Bañuls L., Navarro-García M.M., Escribano A. (2021). New Laboratory Protocol to Determine the Oxidative Stress Profile of Human Nasal Epithelial Cells Using Flow Cytometry. J. Clin. Med..

[13] Rao X., Huang X., Zhou Z., Lin X. (2013). An Improvement of the 2ˆ(-Delta Delta CT) Method for Quantitative Real-Time Polymerase Chain Reaction Data Analysis. Biostat Bioinform Biomath..

[14] Manda-Handzlik A., Demkow U. (2015). Neutrophils: The Role of Oxidative and Nitrosative Stress in Health and Disease. Adv. Exp. Med. Biol..

[15] Janciauskiene S., Welte T. (2016). Well-Known and Less Well-Known Functions of Alpha-1 Antitrypsin. Its Role in Chronic Obstructive Pulmonary Disease and Other Disease Developments. Ann. Am. Thorac. Soc..

[16] Ertel W., Morrison M.H., Ayala A., Chaudry I.H. (1995). Hypoxemia in the Absence of Blood Loss or Significant Hypotension Causes Inflammatory Cytokine Release. Am. J. Physiol.-Regul. Integr. Comp. Physiol..

[17] Hartmann G., Tschöp M., Fischer R., Bidlingmaier C., Riepl R., Tschöp K., Hautmann H., Endres S., Toepfer M. (2000). High altitude increases circulating interleukin-6, interleukin-1 receptor antagonist and C-reactive protein. Cytokine.

[18] Han S., Mallampalli R.K. (2015). The Acute Respiratory Distress Syndrome: From Mechanism to Translation. J. Immunol. Baltim. Md. 1950.

[19] Hoenderdos K., Condliffe A. (2013). The Neutrophil in Chronic Obstructive Pulmonary Disease. Am. J. Resp. Cell. Mol..

[20] Iannitti R.G., Casagrande A., Luca A.D., Cunha C., Sorci G., Riuzzi F., Borghi M., Galosi C., Massi-Benedetti C., Oury T.D. (2013). Hypoxia Promotes Danger-Mediated Inflammation via Receptor for Advanced Glycation End Products in Cystic Fibrosis. Am. J. Resp. Crit. Care..

[21] Thompson A.A.R., Binham J., Plant T., Whyte M.K.B., Walmsley S.R. (2013). Hypoxia, the HIF Pathway and Neutrophilic Inflammatory Responses. BiolChem.

[22] Walmsley S.R., Print C., Farahi N., Peyssonnaux C., Johnson R.S., Cramer T., Sobolewski A., Condliffe A.M., Cowburn A.S., Johnson N. (2005). Hypoxia-Induced Neutrophil Survival Is Mediated by HIF-1alpha-Dependent NF-KappaB Activity. J. Exp. Med..

[23] Beckman J.S., Koppenol W.H. (1996). Nitric Oxide, Superoxide, and Peroxynitrite: The Good, the Bad, and Ugly. Am. J. Physiol-cell Ph..

[24] Pacher P., Beckman J.S., Liaudet L. (2007). Nitric Oxide and Peroxynitrite in Health and Disease. Physiol. Rev..

[25] Papp E., Száiraz P., Korcsmáiros T., Csermely P., Papp E., Száiraz P., Korcsmáiros T., Csermely P. (2006). Changes of Endoplasmic Reticulum Chaperone Complexes, Redox State, and Impaired Protein Disulfide Reductase Activity in Misfolding Ai-antitrypsin Transgenic Mice. Faseb. J..

[26] Borzone G.R., Liberona L.F., Bustamante A.P., Saez C.G., Olmos P.R., Vecchiola A., Villagrán A., Serrano C., Reyes T.P. (2009). Differences in Lung Glutathione Metabolism May Account for Rodent Susceptibility in Elastase-Induced Emphysema Development. Am. J. Physiol.-Regul. Integr. Comp. Physiol..

[27] Marcus N.Y., Blomenkamp K., Ahmad M., Teckman J.H. (2012). Oxidative Stress Contributes to Liver Damage in a Murine Model of Alpha-1-Antitrypsin Deficiency. Exp. Biol. Med..

[28] Escribano A., Amor M., Pastor S., Castillo S., Sanz F., Codoñer-Franch P., Dasí F. (2015). Decreased Glutathione and Low Catalase Activity Contribute to Oxidative Stress in Children with α-1 Antitrypsin Deficiency. Thorax.

[29] Escribano A., Pastor S., Reula A., Castillo S., Vicente S., Sanz F., Casas F., Torres M., Fernández-Fabrellas E., Codoñer-Franch P. (2016). Accelerated Telomere Attrition in Children and Teenagers with A1-Antitrypsin Deficiency. Eur. Respir. J..

[30] Dasi F., Pastor S., Reula A., Castillo S., Escribano A. Augmentation Therapy Increases Hydrogen Peroxide Accumulation in Peripheral Blood Mononuclear Cells of AATD Patients. Proceedings of the American Thoracic Society Annual Meeting.

