# Supplementary material for: Hypoxia Enhances Oxidative Stress in Neutrophils from ZZ Alpha-1 Antitrypsin Deficiency Patients
Source: Antioxidants (Basel). 2023 Apr 3;12(4):872. doi: 10.3390/antiox12040872 (PMC10135227; doi:10.3390/antiox12040872)
Supplement: Supplementary file 1 [file antioxidants-12-00872-s001.zip › antioxidants-2293764-supplementary.pdf]

Supplementary Table S1. Summary of flow cytometry methods.

	Probe	Parameter determined	Probe final concentration	Inductor	Death marker	Detector
**ROS/RNS**	**HE**	O_2^-	3.15 μg/mL	PB	DAPI	PE-Cy7
	**H_2DCFDA**	H_2O_2	3.15 μg/mL	t-BHP	DAPI	FITC
	**DHR 123**	$ONOO^-$	250 ng/ml	PB + NOR-1	DAPI	FITC
	**DAF-FMDA**	NO	1.25 μM	NOR-1	DAPI	FITC
**Non-enzymatic antioxidants**	**CMFDA**	Reduced thiols	31.5 nM	t-BHP	DAPI	FITC
	**MCB**	Reduced GSH	0.016 nM	DEM	PI	BV421
**Mitochondrial parameters**	**TMRM**	Mitochondrial Ψm	756 nM	FCCP	DAPI	PE-Cy7
	**MitoSOX**	Mitochondrial O_2^-	800 nM	PB	DAPI	PE-Cy7
**Intracellular Ca^{2+}**	**FLUO-4**	Intracellular Ca^{2+}	625 nM	Ionomycin	DAPI	FITC
**Oxidative damage to biomolecules**	**BODIPY 665/676**	Oxidized/reduced lipid ratio	1.00 μM	t-BHP	DAPI	PE/APC
	**FTC**	Protein carbonylation	1.00 μM	Ac. Ascórbico + $FeSO_4$	DAPI	FITC
**Cell viability**	**DAPI**	Cell death	800 ng/mL	-	-	-
	**PI**	Cell death	8 μg/mL	-	-	-

Supplementary Table S2. Oxidative stress, mitochondrial, intracellular calcium, and oxidative damage parameters of neutrophils isolated from individuals included in the study.

**Variable**	**MM (n=7)**	**MZ (n=31)**	**SZ (n=8)**	**ZZ (n=15)**	**p-value**
**Superoxide anion (O_2^-)**	650.10 ± 250.3	417.60 ± 181.8	238.80 ± 87.47	457.80 ± 111.8	**0.002**
**Hydrogen peroxide (H_2O_2)**	29.10 (23.70-57.60)	25.40 (22.50-63.90)	57.10 (25.70-88.60)	79.05 (36.70-159.0)	**0.002**
**Peroxynitrite ($ONOO^-$)**	240.00 ± 90.00	377.30 ± 51.83	587.50 ± 145.5	882.10 ± 128.00	**<0.0001**
**Nitric oxide (NO)**	36.40 ± 4.19	41.54 ± 4.50	58.48 ± 12.76	64.37 ± 6.06	**0.028**
**Mitochondrial membrane potential ($\Delta\psi_m$)**	985.2 ± 57.04	625.80 ± 62.96	867.00 ± 94.72	717.90 ± 61.43	**0.007**
**Mitochondrial O_2^- (mtO_2^-)**	13.02 ± 3.39	15.03 ± 1.94	14.59 ± 4.33	20.27 ± 1.82	0.197
**Intracellular calcium (iCa^{2+})**	170.60 ± 7.65	170.40 ± 14.46	128.50 ± 22.64	191.70 ± 17.92	0.202
**Oxidized proteins**	14.35 ± 0.85	21.57 ± 1.65	23.18 ± 1.39	23.07 ± 1.71	**0.041**
**Lipid peroxidation**	75.58 ± 5.07	122.00 ± 22.76	169.90 ± 16.77	133.00 ± 14.36	**0.046**

Data are presented as mean ± standard deviation of arbitrary fluorescence units in those cases where the variable follows a normal distribution. Otherwise, the results are expressed as median (range). p-values lower than 0.05 were statistically significant (labeled in bold).

Supplementary Table S3. Enzymatic and non-enzymatic defense mechanisms of neutrophils isolated from individuals included in the study.

Variable	MM (n=7)	MZ (n=31)	SZ (n=8)	ZZ (n=15)	p-value
**Cu/Zn Superoxide dismutase (Cu/Zn SOD)**	1.26 ± 0.10	0.75 ± 0.17	0.49 ± 0.12	0.56 ± 0.11	**0.018**
**Glutathione Reductase (GR)**	0.70 ± 0.07	0.43 ± 0.07	0.64 ± 0.08	0.30 ± 0.05	**0.0004**
**Catalase**	1.35 ± 0.28	2.07 ± 0.31	2.61 ± 0.49	0.75 ± 0.18	0.178
**Mn Superoxide dismutase (Mn-SOD)**	0.95 ± 0.09	0.72 ± 0.06	1.02 ± 0.16	0.81 ±0.09	0.284
**Glutathione peroxidase (GPx)**	1.05 ± 0.13	0.89 ± 0.20	0.92 ± 0.30	2.07 ± 0.49	0.118
**Nuclear erythroid 2-related factor (Nrf2)**	1.08 ± 0.12	0.85 ± 0.11	0.98 ± 0.24	0.76 ± 0.10	0.456
**Reduced glutathione (GSH)**	370.5 (318-383)	475 (284-585)	529 (482-628)	394 (330-574)	**0.037**
**Reduced thiols**	187.80 ± 10.79	216.70 ± 7.51	271.80 ± 23.62	227.40 ± 14.49	**0.014**

Data are presented as mean ± standard deviation of arbitrary fluorescence units in those cases where the variable follows a normal distribution. Otherwise, the results are expressed as median (range). p-values lower than 0.05 were statistically significant (labeled in bold).
